# The power of cooperation: Experimental and computational approaches in the functional characterization of bacterial sRNAs

**DOI:** 10.1111/mmi.14420

**Published:** 2019-11-28

**Authors:** Jens Georg, David Lalaouna, Shengwei Hou, Steffen C. Lott, Isabelle Caldelari, Stefano Marzi, Wolfgang R. Hess, Pascale Romby

**Affiliations:** ^1^ Faculty of Biology, Genetics and Experimental Bioinformatics University of Freiburg Freiburg Germany; ^2^ Architecture et Réactivité de l’ARN CNRS Université de Strasbourg Strasbourg France; ^3^ Freiburg Institute for Advanced Studies University of Freiburg Freiburg Germany; ^4^ Present address: Department of Biological Sciences University of Southern California Los Angeles CA USA

**Keywords:** CopraRNA, MAPS, post-transcriptional regulation, sRNAs, *Staphylococcus aureus*

## Abstract

Trans‐acting small regulatory RNAs (sRNAs) are key players in the regulation of gene expression in bacteria. There are hundreds of different sRNAs in a typical bacterium, which in contrast to eukaryotic microRNAs are more heterogeneous in length, sequence composition, and secondary structure. The vast majority of sRNAs function post‐transcriptionally by binding to other RNAs (mRNAs, sRNAs) through rather short regions of imperfect sequence complementarity. Besides, every single sRNA may interact with dozens of different target RNAs and impact gene expression either negatively or positively. These facts contributed to the view that the entirety of the regulatory targets of a given sRNA, its targetome, is challenging to identify. However, recent developments show that a more comprehensive sRNAs targetome can be achieved through the combination of experimental and computational approaches. Here, we give a short introduction into these methods followed by a description of two sRNAs, RyhB, and RsaA, to illustrate the particular strengths and weaknesses of these approaches in more details. RyhB is an sRNA involved in iron homeostasis in Enterobacteriaceae, while RsaA is a modulator of virulence in *Staphylococcus aureus*. Using such a combined strategy, a better appreciation of the sRNA‐dependent regulatory networks is now attainable.

## Graphical abstract

The joint application of experimental and the computational tool CopraRNA can provide a comprehensive appreciation of a bacterial sRNA targetome.

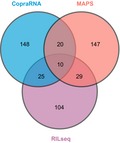

## 1 THE CHALLENGE OF IDENTIFYING THE REGULATORY TARGETS OF BACTERIAL sRNAs

Bacteria experience various metabolic and stress conditions they need to respond rapidly. Small trans‐acting regulatory RNAs (sRNAs), which frequently but not always are noncoding, have been found at the heart of regulatory pathways that allow bacteria to regulate virulence gene expression, respond to stresses, sense the population density, modulate the cell surface composition, and adjust their metabolism (Carrier, Lalaouna, & Massé, 2018; Desgranges, Marzi, Moreau, Romby, & Caldelari, 2019; Holmqvist & Wagner, 2017; Radoshevich & Cossart, 2018). Therefore, it is of utmost interest to identify the RNA targets of these sRNAs, as many bacteria express hundreds of different sRNAs from differentially regulated genes. These sRNAs are very heterogeneous in length, sequence composition, and secondary structure. They can originate from their own genes or may be processed from the 5′ or 3′ UTRs of protein‐coding genes (Chao et al., 2017; Lalaouna et al., 2019). Some sRNAs, such as ArcZ in *Escherichia coli* (Mandin & Gottesman, 2010) or RprA in *Salmonella enterica* (Papenfort, Espinosa, Casadesus, & Vogel, 2015) are even further processed by RNase E, which resulted in different sRNA fragments. Although this processing is essential for the sRNA regulatory functions, it is not yet known whether the targets set of the sRNA is changed (Chao et al., 2017). In *Bacillus subtilis*, it has been described that an additional RNase Y‐dependent processing of RoxS expanded the repertoire of its target mRNAs (Durand et al., 2015).

These sRNAs may not only interact with mRNAs but also with other sRNAs, tRNA precursors, or with proteins. However, the largest class of bacterial sRNAs frequently targets different mRNAs that are often functionally related. The sRNA–mRNA interaction relies on base pairings (including Watson–Crick and G–U) between complementary sequences stretches in the two molecules. To complicate things further, these RNA sequence elements involve usually short (between 8 and less than 50 nts) and noncontinuous base pairings, and can be involved in alternative intramolecular secondary structures competing with the intermolecular interaction. Moreover, interacting proteins such as the RNA chaperones Hfq (Dos Santos, Arraiano, & Andrade, 2019) and ProQ (Smirnov et al., 2016) frequently mediate these interactions in Enterobacteriaceae (Holmqvist & Vogel, 2018). All these factors make the simple bioinformatic search for complementary segments in a given sRNA versus the pool of coding sequences inadequate (Backofen & Hess, 2010). In this review, we have chosen two case studies from the two distant bacteria, *E. coli* and *Staphylococcus aureus*, which have evolved different helper proteins and ribonucleases associated with sRNA regulation.

## 2 DIFFERENT EXPERIMENTAL APPROACHES FOR THE IDENTIFICATION OF sRNA TARGETS

Due to the shortcomings of simple bioinformatic searches, several new experimental RNA‐seq approaches have been developed to enrich and identify the targetome of an sRNA (for a review, see (Saliba, C Santos, & Vogel, 2017)). Here, we will primarily focus on MAPS (MS2‐affinity purification coupled with RNA sequencing) and RIL‐seq (RNA interaction by ligation and sequencing).

In MAPS, the sRNA of interest is tagged usually at its 5′ end with the MS2 RNA aptamer and expressed in bacteria. Cytoplasmic extracts are purified by affinity chromatography followed by the sequencing of the enriched MS2–sRNA–RNA complexes (Lalaouna, Prévost, Eyraud, & Massé, 2017; Lalaouna, Desgranges, Caldelari, & Marzi, 2018). The main advantages of this approach include the sensitive detection of poorly expressed targets, the discrimination between direct and indirect targets, and finding sRNA targets that form base pairings (Lalaouna, Carrier, et al., 2015). The approach was applied successfully to many sRNAs from Gram‐negative bacteria such as *E. coli* and *Salmonella* Typhimurium, and from Gram‐positive bacteria like *S. aureus* (see below).

To achieve a genome wide analysis of sRNA–RNA interactions, both CLASH (cross linking, ligation, and sequencing of hybrids) and RIL‐seq rely on the association of sRNAs–targets with RNA‐binding proteins (Melamed et al., 2016; Waters et al., 2017). In this application of the CLASH method, sRNA–target hybrids bound by RNase E were explored (Waters et al., 2017), while RIL‐seq investigated interactions of RNAs associated with the RNA chaperone Hfq (Melamed et al., 2016). In both methods, the tagged proteins and the bound RNAs are purified by affinity chromatography, then the RNA hybrids are ligated and the resulting RNA chimeras are sequenced. RIL‐seq has expanded the ensemble of known targets of sRNAs in *E. coli* and showed the dynamics of the regulatory networks under various stress conditions (Melamed et al., 2016). These two interactome methods are not limited to a specific sRNA and can simultaneously identify a great number of RNA–RNA interactions. The RIL‐seq approach has been restricted until now to *E. coli* as Hfq in several Gram‐positive bacteria has limited RNA chaperone activity (Zheng, Panja, & Woodson, 2016). Only in *Listeria monocytogenes*, Hfq has been found as a key partner of sRNAs (Nielsen et al., 2010). As *Listeria* expresses numerous sRNAs involved in virulence (Cerutti et al., 2017; Toledo‐Arana et al., 2009), the RIL‐Seq approach using Hfq might be an appropriate strategy to simultaneously probe RNA–sRNA interactions involved in stress tolerance and virulence, conditions that require Hfq (Nielsen et al., 2010).

## 3 ADVANCED ALGORITHMIC APPROACHES IN THE IDENTIFICATION OF sRNA TARGETS

Computational RNA–RNA interaction prediction methods could be a fast and cheap alternative or complement to the experimental approaches. These tools (reviewed in Wright, Mann, & Backofen, 2018) use a thermodynamic model of base pairing to find stretches of complementary bases in two RNA sequences that can form a stable intermolecular duplex. A major feature of more advanced methods is the consideration of the interaction site accessibility, that is, the question of whether intramolecular structures within sRNA and target RNA interfere with the intermolecular duplex formation (Backofen & Hess, 2010). There are two distinct tasks for bioinformatic tools: (a) finding the actual target site, that is, the interacting bases, for a given sRNA–target pair that was discovered experimentally. This site can be subsequently scrutinized by additional experiments, such as reporter gene assays in combination with point mutations in the predicted interaction regions of the mRNA and sRNA. (b) Predicting the full targetome of a given sRNA. In practice, this means to predict the best possible interactions between the given sRNA and all possible target sequences in the respective transcriptome. Then, the results can be ranked by the minimal energies of the predicted interactions and true positives should appear higher than false positives.

Tools like IntaRNA (Busch, Richter, & Backofen, 2008) or RNAplex (Tafer & Hofacker, 2008) are quite successful to cope with the first task. For instance, the true positive rate and the positive predictive value for the prediction of base pairs in 109 experimentally verified interactions is 0.62 and 0.64 for IntaRNA, respectively (Lai & Meyer, 2016). However, all existing noncomparative tools struggle more or less with the second task, the targetome prediction (Pain et al., 2015). To improve the computational performance for the full genome target prediction, the comparative CopraRNA algorithm was developed (Wright et al., 2014, 2013). The general principle of CopraRNA can be described as follows: if an sRNA is conserved in different organisms, also the targets should be conserved to some extent. Thus, a true positive interaction should appear in multiple organisms, while a false positive interaction should be restricted to a single prediction. Simply, CopraRNA joins a set of organism‐specific target predictions by IntaRNA into a combined prediction, which leads to an increased sensitivity and specificity. Indeed, a separate benchmarking study revealed that CopraRNA currently provides the best and most advanced computational strategy to predict bacterial sRNA targets (Pain et al., 2015). Furthermore, it was reported that CopraRNA has only a slightly worse specificity (2.7% vs. 4.7%) and sensitivity (52.7% vs. 53.4%) than the experimental RIL‐seq method (Melamed et al., 2016).

CopraRNA not only produces a list of likely targets but also generates a complex set of additional information. The algorithm defines the regulatory domains of the sRNAs, performs functional enrichment of the predicted target mRNAs, and reconstructs the regulatory networks (Wright et al., 2014, 2013). Originally, CopraRNA was successfully tested on 18 well‐known Enterobacteriaceae sRNAs and on a small number of sRNAs from different bacteria revealing the previously identified mRNA targets as well as novel base pairing interactions (Wright et al., 2013). In the following, multiple studies confirmed the power of such approach in diverse bacterial groups including, for example, *Cyanobacteria* (*Synechocystis*: PsrR1 (Georg et al., 2014), NsiR4 (Klähn et al., 2015), IsaR1 (Georg et al., 2017); *Anabaena*: NsrR1 (Álvarez‐Escribano, Vioque, & Muro‐Pastor, 2018, p. 1)), Firmicutes (*Bacillus*: RsaE (Durand et al., 2015)), γ‐Proteobacteria (*Pseudomonas*: RgsA (Lu et al., 2016)), α‐Proteobacteria (*Agrobacterium*: AbcR1 (Overlöper et al., 2014); *Sinorhizobium*: EcpR1, GspR (Robledo et al., 2018)), or β‐Proteobacteria (*Burkholderia*: ncS35, *Neisseria*: FnrS (Kiekens, Sass, Van Nieuwerburgh, Deforce, & Coenye, 2018; Wright et al., 2013)).

## 4 STRENGTHS AND WEAKNESSES OF DIFFERENT METHODS

Despite the excellent performance of the leading experimental (RIL‐seq, MAPS) and computational (CopraRNA) methods, each individual approach nevertheless suffers from false positives (specificity) and struggles to recover the full targetome of a given sRNA leading to false negatives (sensitivity). Each method has its individual strengths and weaknesses.

### 4.1 False positives

In case of computational predictions the tested sequence space is high, while the actual sRNA–target interactions are often short and involve noncontinuous base pairings. This leads to a large number of random interactions with similar or even lower minimal interaction energies than the true targets. This is the main reason for the lower performance of noncomparative bioinformatic tools and the resulting high number of false positives. While this problem has been resolved by considering comparative information, several examples studied in more detail have shown that the problem of false positives is often overrated due to incomplete knowledge about the “true” targetome of any given sRNA. This is because all predicted “true” target until they are independently validated, will be considered as false positive. Furthermore, a predicted (mRNA) target can act as a “sponge” that sequesters the sRNA to inhibit interactions with other targets (Figueroa‐Bossi, Valentini, Malleret, Fiorini, & Bossi, 2009; Grüll & Massé, 2019; Miyakoshi, Chao, & Vogel, 2015). In this scenario, the mRNA is actually the regulator of the sRNA, which hinders the use of reporter gene assay for validation. For experimental in vivo methods such as MAPS or RIL‐seq, the source of false positives is less obvious. Both methods require an actual physical interaction of the sRNA and the targets. However, unspecific binding to any molecule involved in the respective enrichment protocols other than the sRNA cannot be excluded (e.g., MS2 aptamer, column, beads). The RIL‐seq protocol includes a ligation step of the RNA heteroduplexes, which ensures that only RNAs that have been in close proximity are identified as interaction partners. MAPS interprets all RNAs that are purified by the MS2 tagged sRNA as interaction partners. However, indirect copurification with RNA binding proteins (e.g., Hfq, ProQ, or CsrA in Enterobacteriaceae) or with other RNAs might occur. An example would be an mRNA which is targeted by the MS2‐tagged sRNA and also by another sRNA. RIL‐seq uses a crosslinking step, which might lead to artifacts for molecules that are not directly interacting, but that are nevertheless in close vicinity in the cell.

### 4.2 False negatives

The other side of the coin is the biologically “true” targets that are not detected by the respective method.

In case of CopraRNA, there are two major reasons for false negatives. The first reason is linked to the target sequence or the interaction region, which is not in the set of sequences that are scanned for interactions. Indeed, the searched sequence space was reduced to the most likely interaction regions, that is, the 5′UTR (often 200 nts upstream of the annotated start codon) and the first 100 nts in the coding region. This heuristic reduces the number of random, potential false positive, high‐ranking predictions, but also prevents the detection of some targets. In many bacteria, majority of 5′UTRs is only 20–40 nucleotides long (Kim et al., 2012; Kopf et al., 2014; Ruiz de los Mozos et al., 2013; Voigt et al., 2014), and therefore, CopraRNA could potentially predict interactions with nontranscribed sequences. Other mRNAs possess very long 5′UTRs (e.g., ~570 nt for *rpoS* in *E. coli* and ~410 nt for *sarA* in *S. aureus*). Thus, part of these 5′UTRs is overlooked. In the same vein, the coding sequence beyond the first 100 nt after the first nucleotide of the start codon and the 3′UTR are not taken into account, and some sRNAs are indeed pairing deep in the coding sequence (Gutierrez et al., 2013; Lalaouna, Morissette, Carrier, & Massé, 2015; Papenfort, Sun, Miyakoshi, Vanderpool, & Vogel, 2013). Finally, CopraRNA concentrates on protein‐coding mRNAs, that is, noncoding transcripts such as other sRNAs or tRNAs are not yet considered. This should not be neglected since interactions between sRNAs (e.g., RsaI and RsaG (Bronesky et al., 2019)) and between sRNAs and tRNA precursors (e.g., RyhB and 3′ETS*^leuZ^* (Lalaouna, Carrier, et al., 2015)) have been revealed first by MAPS technology, as well as by RIL‐seq, and CLASH (reviewed in Grüll & Massé, 2019). The second reason for false negatives by CopraRNA is that the in silico predicted minimal interaction energy might not reflect the energy value of an in vivo setting. For some targets the strength of intramolecular interactions might be overrated in the prediction, because in vivo these internal structures may sometimes be disrupted by RNA‐binding proteins such as Hfq, ProQ, or CsrA in Enterobacteriaceae (Dos Santos et al., 2019; Müller, Gimpel, Wildenhain, & Brantl, 2019; Smirnov et al., 2016). Furthermore, discontinuous interactions such as double kissing complexes, for example, between OxyS and *fhlA* in *E. coli* (Argaman & Altuvia, 2000) or between RNAIII and *rot* mRNA in *S. aureus* (Romilly et al., 2012) cannot be predicted.

In the experimental targetome approaches, a major reason for false negatives is surely that the respective target is not or only weakly expressed in the used experimental setting. An exhaustive targetome search would thus require the repetition of the experiment with multiple relevant stress and growth conditions. Consequently, the individual results should be rather considered as a snapshot of dynamic events. Additionally, both RIL‐seq and CLASH cannot uncover RNA interactions that are independent of the respective RNA binding protein used for the pulldown of the RNA–RNA heteroduplexes. To identify only directly interacting partners, RIL‐seq and CLASH use a crosslinking and a ligation step. However, their efficiency is pretty low, leading to the loss of a significant number of real RNA:RNA interactions. In case of MAPS, it cannot be excluded that the used MS2 RNA tag interferes with the secondary structure of the tested sRNA and the interaction to some targets. Furthermore, for MAPS some (weaker) targets might be lost during the affinity purification and clean up steps of the protocol. In Enterobacteriaceae, many sRNA‐dependent repressions lead to the rapid depletion of the target mRNAs. These mRNAs might be underestimated during the MAPS purification step.

Besides these fundamental aspects, each method has unique characteristics. Within its limitations, RIL‐seq reveals the Hfq‐dependent interactome of an organism with all detectable RNA–RNA interactions simultaneously. In contrast, MAPS deeply scrutinizes the targetome of a specific sRNA, regardless of the dependency on auxiliary proteins such as Hfq. CopraRNA stands in between, each individual prediction covers only a single sRNA, but it is easy to use CopraRNA as a workflow for multiple sRNAs in parallel. CopraRNA can be also used for genetically intractable organisms. Though, the comparative nature of CopraRNA requires that homologs of the respective sRNA are annotated in the sequence databases. Also the evolutionary distance of the respective genomes should be not too close or too far, with the optimal method to compose the best combination of organisms still being a subject of investigation. Finally, in contrast to experimental methods which involve molecular cloning, bacterial growth (at different conditions), RNA extraction, enrichment and processing steps, library preparation, RNA‐seq, and bioinformatic data analysis, CopraRNA only requires the genomic information, is free of charge and the results are available within a few hours of computation time. Due to the individual strengths of the discussed approaches, it seems highly beneficial to combine different methods for a comprehensive picture of an sRNA regulon. In the following, we exemplify how the results of MAPS, RIL‐seq, and CopraRNA for the *E. coli* sRNA RyhB, and MAPS and CopraRNA for the *S. aureus* sRNA RsaA can be integrated, to test this assumption. Of note, *S. aureus* is the only Gram‐positive bacterium for which the full targetome of a specific sRNA was defined (Bronesky et al., 2019; Lalaouna et al., 2019; Rochat et al., 2018; Tomasini et al., 2017).

## 5 THE POWER OF COOPERATION: INTEGRATION OF EXPERIMENTAL AND COMPUTATIONAL APPROACHES

### 5.1 RyhB, an sRNA involved in iron homeostasis

The first test case was the well‐characterized sRNA RyhB, involved in iron homeostasis in *E. coli*. The analysis of RyhB was initially used as a proof of principle for the MAPS technology (Lalaouna, Carrier, et al., 2015), which has demonstrated to be very effective, as emphasized by the copurification of previously known targets like *sodB*, *sdhC,* and *shiA* mRNAs (Massé & Gottesman, 2002; Prévost et al., 2007; Vecerek, Moll, Afonyushkin, Kaberdin, & Bläsi, 2003), but also by the discovery of the 3′ETS*^leuZ^*, a tRNA‐derived fragment acting as a sRNA sponge.

**Figure 1:**
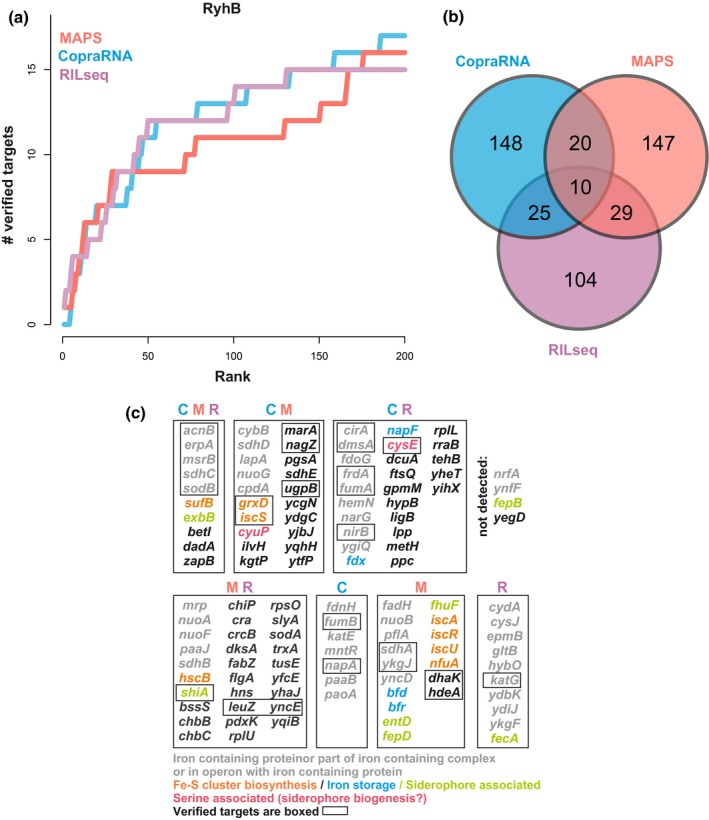
Comparison of MAPS, CopraRNA, and RIL‐seq data to characterize the RyhB targetome. The MAPS and RIL‐seq data were taken from references (Lalaouna, Carrier, et al., 2015) and (Melamed et al., 2016), respectively. (a) The cumulated number of well‐characterized targets are plotted against the prediction rank for CopraRNA, MAPS, and RIL‐seq as an estimate of sensitivity and specificity. (b) The Venn diagram shows the overlap of the top‐200 MAPS, RIL‐seq, and CopraRNA predictions. (c) Gene names are classified into subgroups according to the Venn diagram (detected by multiple methods or unique to one method) but also to their functional role or link to iron homeostasis. Genes in black characters are not directly linked to iron. C is for CopraRNA, M for MAPS, and R for RIL‐seq

We compared the top 200 results taken from a slightly modified CopraRNA version and from MAPS. To complete the analyses, we added the results from the global Hfq‐dependent RIL‐seq interactome study with RyhB (Melamed et al., 2016) and used a set of 30 independently verified RyhB targets as reference (Figure 1a). The benchmark shows that the methods are highly complementary (Figure 1b) since only 10/30 targets are detected by all methods, while 4/30 targets are not predicted by any. Moreover, 18, 17, and 15 verified targets are in the top lists of CopraRNA, MAPS, and RIL‐seq, with 2, 4, and 1 uniquely identified targets, respectively. Unsurprisingly, the top targets were highly enriched in mRNAs for iron‐binding proteins or proteins related to iron homeostasis (iron transport, siderophore, and Fe/S cluster biosynthesis, iron storage; Figure 1c), with the majority of those not yet independently verified or described as RyhB targets (48/67). Among the candidates, 10 of them are detected by all methods, including 5 well‐characterized targets and 2 iron‐related putative targets (*sufB* and *exbB*). Only *betI* (transcriptional regulator), *dadA* (d‐aminoacid dehydrogenase) and *zapB* (cell division protein) mRNAs are not clearly related to iron.

It is hard to pin down the reasons for individual true targets not being detected by one or the other method. However, there is a likely explanation for some of them. For instance, MS2‐RyhB MAPS was performed in exponential phase of growth (OD_600nm_ = 0.5) in LB‐rich medium. Knowing that RyhB is mainly produced in response to iron starvation, some targets are weakly or not expressed under the tested conditions (e.g., *nirB* mRNA (Massé, Vanderpool, & Gottesman, 2005)). Therefore, while *nirB* could not be found using MAPS, CopraRNA successfully identified *nirB* as one of the best candidates regardless of its expression (Wright et al., 2013). Later on, *nirB* was also detected by RIL‐seq, which was done at various conditions including iron starvation (Melamed et al., 2016). As mentioned above, the noncoding tRNA precursor *leuZ–glyW–cysT* was missed in the CopraRNA search space. Finally, for the *yncE* and *yegD* mRNAs, they belong to the cases were the computationally predicted interaction energy was not good enough to secure a place in the top list. The predicted interaction energies in *E. coli* are only ~−5 kcal/mol and ~6 kcal/mol, putting *yncE* and *yegD* at rank 834 and 2,422 in the list of CopraRNA results. In these cases, the predicted energy most likely diverges from the actual energy in a living cell.

For MAPS, stable interactions like overlapping UTRs of mRNAs (numerous genes in the top list are encoded tail to tail or head to head; *fepD/entS*, *yccX/tusE*, *ycgJ/pliG*, *yncD/yncE*, *ynfA/ynfB*, *patA/ygjH*, *fadH/higA*) or between mRNAs and sRNAs (*chbBC*:ChiX sRNA; (Figueroa‐Bossi et al., 2009)) could be indirectly copurified when one of the partners was targeted by the MS2‐tagged sRNA. Another aspect needs to be considered when using MAPS. The whole operon transcripts are commonly copurified, while CopraRNA and RIL‐seq only focus on the localization of the interaction site. Therefore, several candidates identified by MAPS are part of an operon targeted by RyhB (e.g., *iscRSUA* or *glyW–cysT–leuZ*). In most cases, they should be considered as true targets as RyhB pairing commonly induces the decay of the entire transcript. The only exception is the *glyW–cysT–leuZ* operon, which is the precursor of the 3′ETS*^leuZ^* and is not regulated by RyhB (Lalaouna, Carrier, et al., 2015).

### 5.2 RsaA, an sRNA that modulates virulence

RsaA is a sigma B‐dependent regulatory RNA identified in *S. aureus* as an attenuator of acute infection and promoting chronic diseases (Romilly et al., 2014). Transcription of the *rsaA* gene leads to the synthesis of a short form (138 nts) and a long form (286 nts) most probably due to transcriptional readthrough. The longest form is less well expressed and appears to be less stable than the short version of RsaA (Geissmann et al., 2009). Using different approaches (proteomic and MAPS), the mRNA encoding the posttranscriptional repressor MgrA was characterized as the main target of RsaA (Romilly et al., 2014; Tomasini et al., 2017). By repressing *mgrA*, RsaA inhibits capsule formation and induces biofilm formation. In addition, MAPS allowed the identification of four other targets such as the SsaA‐like enzymes (i.e., *ssaA_2*: #14, *ssaA2_2*: #6, and *ssaA2_3*: #3) involved in peptidoglycan metabolism, and the anti‐inflammatory FLIPr protein (*flr*: #12). In the same study, quantitative proteomic experiments revealed that the synthesis of several cell surface proteins was activated by RsaA as the results of *mgrA* repression (Tomasini et al., 2017).

**Figure 2:**
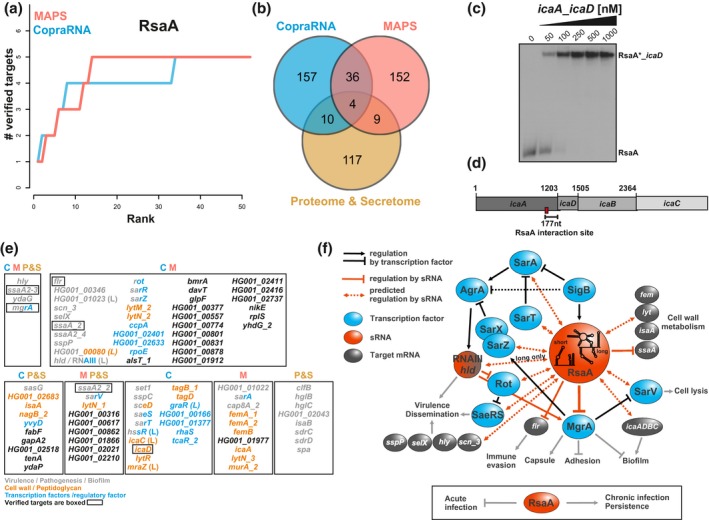
Integration of MAPS, CopraRNA, and proteomic data to estimate the RsaA targetome. The MAPS and proteomic data were taken from reference (Tomasini et al., 2017). (a) The cumulated numbers of known targets are plotted against the prediction rank for CopraRNA and MAPS as an estimate of sensitivity and specificity. (b) The Venn diagram shows the overlap of the top 200 MAPS and CopraRNA predictions and the proteins, which were significantly differentially synthesized in the proteome and secretome data. (c) Gel retardation assays using the short version of RsaA and a putative mRNA target. 5′end‐radiolabeled RsaA(*) was incubated with increasing concentrations of *icaA*_*icaD* fragment (nts −239 to +175, from the AUG of *icaD*). (d) Schematic visualization of the *icaADBC* operon and the location of the verified interaction site with RsaA. (e) Gene names or locus tags of the genes that were detected by more than one method or targets that are unique to one method but belong to one of the indicated functional categories. Genes in black characters encode hypothetical proteins or proteins with functions unrelated to the major targets of RsaA. C is for CopraRNA, M for MAPS, P for proteome, and S for secretome. (f) Schematic drawing of the regulatory networks involving RsaA. The experimental data are taken from Romilly et al. (2014) and Tomasini et al. (2017). Black arrows (activation) or bars (repression) are for transcriptional control, red arrows (activation) or bars (repression) are for post‐transcriptional control. Transcriptional regulators are in blue, regulatory RNAs are in red and other mRNAs regulated by RsaA are in grey. Dashed arrows are for potential RsaA targets for which experimental validation is required

Then, we wondered what we could learn beyond the already known targets by the combination of MAPS and CopraRNA. Therefore, we compared the top 200 results of MAPS and the hits obtained with a slightly modified version of CopraRNA using RsaA short or long as input (Figure 2a). Remarkably, *mgrA* mRNA was the number one target in the CopraRNA prediction. Three of the four additional targets were in the top 34 prediction of CopraRNA too (*ssaA2_3*: #6, *ssaA_2*: #8, *flr* #34). The performance of both methods regarding known targets is shown in Figure 2a. We also added the genes, which were identified by the differential proteomic analysis in the reference (Tomasini et al., 2017) (Figure 2b). The Venn diagram in Figure 2b shows the overlap of the three datasets. Targets that appeared in more than one dataset are of special interest, as they are more likely true targets. Four targets appear in all three datasets. These are the already known *mgrA* and *ssaA2–3* mRNAs, and the so far not independently verified mRNAs encoding the hemolysin α (*hly*) and the general stress protein YdaG.

Functional enrichment (Huang, Sherman, & Lempicki, 2009) revealed that the overlapping target set and also the top 200 individual target sets are highly enriched in genes encoding virulence factors and secreted proteins. The preceding studies on RsaA reported also its involvement in the regulation of peptidoglycan metabolism and biofilm formation (Romilly et al., 2014; Tomasini et al., 2017). Uniquely predicted targets with a high rank, belonging to one of these categories (Figure 2c) are also potential additional true targets missed by the experimental methods. MAPS also detected the sRNA RNAIII, the quorum sensing induced regulatory RNA, as a potential interaction partner of RsaA. RNAIII is a dual‐function sRNA that also codes for hemolysin δ (*hld*), which is a CopraRNA‐predicted target of the long form of RsaA. Because the yield of RNAIII does not change significantly in a mutant ∆*rsaA* strain, it remains to be analyzed whether RsaA might regulate *hld* translation as suggested by the CopraRNA prediction. In addition to *mgrA*, we also observed several virulence associated regulatory proteins (i.e., Rot, SarR, SarZ (CopraRNA & MAPS); SarV (MAPS & proteome); SarA (MAPS)) although most of their mRNAs were only poorly enriched with MS2‐tagged RsaA. Noteworthy, several of these potential RsaA‐dependent targets are directly connected to MgrA regulon. For instance, MgrA acts both as a transcriptional repressor of SarV, a regulator of autolysis, and as a transcriptional activator of SarX and SarZ, two negative regulators of the *agr* system (Figure 2f; Cheung, Nishina, Trotonda, & Tamber, 2008). The RsaA‐dependent regulation of the expression of enzymes involved in cell wall metabolism and integrity, in adhesion and biofilm formation, in capsule formation are certainly part of complex regulatory networks favoring harmful colonization and chronic infections in human (Figure 2f; Romilly et al., 2014; Tomasini et al., 2017)).

Returning to method‐specific characteristics, we will here address selected errors and shortcomings of MAPS and CopraRNA. Because it is a difficult task to detect false positives, we concentrate on targets that are missed by one or the other method (false negatives). Based on the rank 2 CopraRNA predictions, RsaA could interact with the 3′ end of *icaA* (*icaD* in Figure 2c). Using gel retardation assays, we validated the formation of an efficient and stable complex between RsaA and *icaA–icaD* mRNA fragment (nucleotides −239 to +175, from the AUG of *icaD*; Figure 2d,e). However, the polycistronic *icaADBC* mRNA was not considered in the first conservative analysis of the MS2‐RsaA MAPS data due to the relatively low enrichment factor (*icaA* (FC 1.94, rank 28); *icaD* (FC 1.14)). The reason for this low enrichment is certainly due to a poor expression of *icaADBC* operon in tested conditions, as validated by transcriptomic analysis (Tomasini et al., 2017). CopraRNA in turn did not predict *ssaA2_2* mRNA due to a straightforward reason. Prior to the combination of predictions from different organisms, CopraRNA needs to identify which mRNAs code for homologous proteins. This is done using the DomClust algorithm (Uchiyama, 2006). The two proteins SsaA2_2 and SsaA_2 are too similar (76.7% sequence identity) to be assigned to different clusters. In the following, only *ssaA_2* with the best predicted interaction energy of −14.49 kcal/mol, and not *ssaA2_2* (−11.97 kcal/mol), was considered for the combination. Using the poor *ssaA2_2* prediction would have resulted also in prediction rank 10.

## 6 CONCLUSION

### 6.1 The power of cooperation

As a surprising outcome of the present analysis, we found that the general performance of the methods MAPS, CopraRNA and RIL‐seq with respect to sensitivity and specificity are largely comparable, but that the overlap between each set of predictions is remarkably low. Due to the different strengths and weaknesses, no single experimental or computational approach can uncover all targets of an sRNA alone. The combination of conceptually independent approaches should drastically enhance the specificity because the different individual reasons for possible false‐positive prediction are unlikely to coincide, with the choice of the experimental method being highly dependent on the bacterial model. However, we have not verified this assumption here, as it would require the independent validation of all predicted targets in the intersection of the presented methods (Figures 1b and 2b).

### 6.2 New findings due to cooperation

Combining the results of the different methods for the two sRNAs chosen as examples yielded several interesting new findings.

With more than 15 years research and numerous scientific studies, RyhB is well established as a regulator of iron homeostasis in *E. coli* and other *γ‐*proteobacteria and presumably one of the best investigated bacterial sRNAs overall. Nevertheless, the combined CopraRNA, MAPS, and RIL‐seq data indicate that the RyhB regulon is significantly larger than described so far (Figure 1). In total, there are more than 30 suggested new targets, including the mRNAs of multiple iron containing proteins (e.g., *napF*, *fdx*), mRNAs with a clear link to iron import (e.g., *exbB*, *fepD*), and *cyuP* coding for a serine transporter, which is relevant for siderophore biosynthesis.

The sRNA RsaA is a key modulator of *S. aureus* virulence (Romilly et al., 2014; Tomasini et al., 2017). Besides MgrA, the major regulator of capsule formation, RsaA might also affect a multitude of major virulence regulatory systems. This includes the transcription factors of the Sar family (Rot, SarA, SarT, SarR, SarV, SarZ), the regulatory RNAIII, and several virulence associated secreted proteins or surface proteins. These factors include the hemolysin α (*hly*), the enterotoxin‐like toxin X (*selX*), a complement inhibitor (*scn_3*), the surface protein G (*sasG*), and staphopain A (*sspP*). The third large group of predicted targets copes with the peptidoglycan/cell wall metabolism and biofilm formation. Examples are the *icaADBC* operon and the cell wall hydrolases. This study also indicates that the longest form of RsaA, even less expressed, regulates additional targets, expanding the regulon of RsaA (Figure 2). Even though these predictions still await independent validation, the combined results of MAPS and CopraRNA provide a promising basis for further investigations. This strategy is particularly useful in organisms where no major RNA‐binding proteins have yet been found associated with sRNA such as in Gram‐positive bacteria.

These new findings nicely illustrate what can be gained from the joint application of experimental and computational approaches on defining the targetome of a bacterial sRNA, a prerequisite to better decipher its roles in bacteria physiology, stress response, adaptation, and virulence. A major challenge will be to adapt these new methods to probe the dynamics of RNA–RNA interactions in more complexed ecological environments and in *in vivo* models of infection for pathogenic bacteria.

## CONFLICT OF INTEREST

The authors have no conflict of interest to declare.

## References

[mmi14420-bib-0001] Álvarez‐Escribano, I. , Vioque, A. , & Muro‐Pastor, A. M. (2018). NsrR1, a nitrogen stress‐repressed sRNA, contributes to the regulation of *nblA* in *Nostoc* sp. PCC 7120. Frontiers in Microbiology, 9, 2267 10.3389/fmicb.2018.02267 30319578 PMC6166021

[mmi14420-bib-0002] Argaman, L. , & Altuvia, S. (2000). fhlA repression by OxyS RNA: Kissing complex formation at two sites results in a stable antisense‐target RNA complex. Journal of Molecular Biology, 300, 1101–1112. 10903857 10.1006/jmbi.2000.3942

[mmi14420-bib-0003] Backofen, R. , & Hess, W. R. (2010), Computational prediction of sRNAs and their targets in bacteria. RNA Biology, 7, 33–42. 10.4161/rna.7.1.10655 20061798

[mmi14420-bib-0004] Bronesky, D. , Desgranges, E. , Corvaglia, A. , François, P. , Caballero, C.J. , Prado, L. , … Marzi, S. (2019). A multifaceted small RNA modulates gene expression upon glucose limitation in *Staphylococcus aureus* . EMBO Journal, 38, pii: e99363 10.15252/embj.201899363 30760492 PMC6418428

[mmi14420-bib-0005] Busch, A. , Richter, A. S. , & Backofen, R. (2008). IntaRNA: Efficient prediction of bacterial sRNA targets incorporating target site accessibility and seed regions. Bioinformatics, 24, 2849–2856. 10.1093/bioinformatics/btn544 18940824 PMC2639303

[mmi14420-bib-0006] Carrier, M.‐C. , Lalaouna, D. , & Massé, E. (2018). Broadening the definition of bacterial small RNAs: Characteristics and mechanisms of action. Annual Review of Microbiology, 72, 141–161. 10.1146/annurev-micro-090817-062607 30200848

[mmi14420-bib-0007] Cerutti, F. , Mallet, L. , Painset, A. , Hoede, C. , Moisan, A. , Bécavin, C. , … Chiapello, H. (2017). Unraveling the evolution and coevolution of small regulatory RNAs and coding genes in Listeria. BMC Genomics, 18, 882 10.1186/s12864-017-4242-0 29145803 PMC5689173

[mmi14420-bib-0008] Chao, Y. , Li, L. , Girodat, D. , Förstner, K. U. , Said, N. , Corcoran, C. , … Luisi, B. F. (2017). In vivo cleavage map illuminates the central role of RNase E in coding and non‐coding RNA pathways. Molecular Cell, 65, 39–51. 28061332 10.1016/j.molcel.2016.11.002 PMC5222698

[mmi14420-bib-0009] Cheung, A. L. , Nishina, K. A. , Trotonda, M. P. , & Tamber, S. (2008). The SarZ protein family of *Staphylococcus aureus* . The International Journal of Biochemistry and Cell Bology, 40, 355–361. 10.1016/j.biocel.2007.10.032 PMC2274939 18083623

[mmi14420-bib-0010] Desgranges, E. , Marzi, S. , Moreau, K. , Romby, P. , & Caldelari, I. (2019). Noncoding RNA. Microbiology Spectrum, 7 10.1128/microbiolspec.GPP3-0038-2018 PMC11590673 31004423

[mmi14420-bib-0011] Dos Santos, R. F. , Arraiano, C. M. , & Andrade, J. M. (2019). New molecular interactions broaden the functions of the RNA chaperone Hfq. Current Genetics, 65(6), 1313–1319. 10.1007/s00294-019-00990-y 31104083

[mmi14420-bib-0012] Durand, S. , Braun, F. , Lioliou, E. , Romilly, C. , Helfer, A.‐C. , Kuhn, L. , … Condon, C. (2015). A nitric oxide regulated small RNA controls expression of genes involved in redox homeostasis in *Bacillus subtilis* . PLoS Genetics, 11, e1004957 10.1371/journal.pgen.1004957 25643072 PMC4409812

[mmi14420-bib-0013] Figueroa‐Bossi, N. , Valentini, M. , Malleret, L. , Fiorini, F. , & Bossi, L. (2009). Caught at its own game: Regulatory small RNA inactivated by an inducible transcript mimicking its target. Genes & Development, 23, 2004–2015. 10.1101/gad.541609 19638370 PMC2751969

[mmi14420-bib-0014] Geissmann, T. , Chevalier, C. , Cros, M. J. , Boisset, S. , Fechter, P. , Noirot, C. , … Romby, P. (2009). A search for small noncoding RNAs in *Staphylococcus aureus* reveals a conserved sequence motif for regulation. Nucleic Acids Research, 37(21), 7239–7257. 10.1093/nar/gkp668 19786493 PMC2790875

[mmi14420-bib-0015] Georg, J. , Dienst, D. , Schurgers, N. , Wallner, T. , Kopp, D. , Stazic, D. , … Wilde, A. (2014). The small regulatory RNA SyR1/PsrR1 controls photosynthetic functions in cyanobacteria. The Plant Cell, 26, 3661–3679. 10.1105/tpc.114.129767 25248550 PMC4213160

[mmi14420-bib-0016] Georg, J. , Kostova, G. , Vuorijoki, L. , Schön, V. , Kadowaki, T. , Huokko, T. , … Hess, W. R. (2017). Acclimation of oxygenic photosynthesis to iron starvation is controlled by the sRNA IsaR1. Current Biology, 27, 1425–1436.e7. 10.1016/j.cub.2017.04.010 28479323

[mmi14420-bib-0017] Grüll, M. P. , & Massé, E. (2019). Mimicry, deception and competition: The life of competing endogenous RNAs. Wiley Interdisciplinary Reviews: RNA, 10(3), e1525 10.1002/wrna.1525 30761752

[mmi14420-bib-0018] Gutierrez, A. , Laureti, L. , Crussard, S. , Abida, H. , Rodríguez‐Rojas, A. , Blázquez, J. , … Matic, I. (2013). β‐lactam antibiotics promote bacterial mutagenesis via an RpoS‐mediated reduction in replication fidelity. Nature Communications, 4, 1610 10.1038/ncomms2607 PMC3615471 23511474

[mmi14420-bib-0019] Holmqvist, E. , & Vogel, J. (2018). RNA‐binding proteins in bacteria. Nature Reviews Microbiology, 16(10), 601–615. 10.1038/s41579-018-0049-5 29995832

[mmi14420-bib-0020] Holmqvist, E. , & Wagner, E. G. H. (2017). Impact of bacterial sRNAs in stress responses. Biochemical Society Transactions, 45, 1203–1212. 10.1042/BST20160363 29101308 PMC5730939

[mmi14420-bib-0021] Huang, D. W. , Sherman, B. T. , & Lempicki, R. A. (2009). Systematic and integrative analysis of large gene lists using DAVID bioinformatics resources. Nature Protocols, 4, 44–57. 10.1038/nprot.2008.211 19131956

[mmi14420-bib-0022] Kiekens, S. , Sass, A. , Van Nieuwerburgh, F. , Deforce, D. , & Coenye, T. (2018). The small RNA ncS35 regulates growth in Burkholderia cenocepacia J2315. mSphere, 3, pii: e00579-17 10.1128/mSphere.00579-17 29359187 PMC5760752

[mmi14420-bib-0023] Kim, D. , Hong, J.‐J. , Qiu, Y. U. , Nagarajan, H. , Seo, J.‐H. , Cho, B.‐K. , … Palsson, B. Ø. (2012). Comparative analysis of regulatory elements between *Escherichia coli* and *Klebsiella pneumoniae* by genome‐wide transcription start site profiling. PLoS Genetics, 8, e1002867 10.1371/journal.pgen.1002867 22912590 PMC3415461

[mmi14420-bib-0024] Klähn, S. , Schaal, C. , Georg, J. , Baumgartner, D. , Knippen, G. , Hagemann, M. , … Hess, W. R. (2015). The sRNA NsiR4 is involved in nitrogen assimilation control in cyanobacteria by targeting glutamine synthetase inactivating factor IF7. Proceedings of the National Academy of Sciences, 112, E6243–E6252. 10.1073/pnas.1508412112 PMC4653137 26494284

[mmi14420-bib-0025] Kopf, M. , Klähn, S. , Scholz, I. , Matthiessen, J. K. F. , Hess, W. R. , & Voß, B. (2014). Comparative analysis of the primary transcriptome of *Synechocystis* sp. PCC 6803. DNA Research, 21, 527–539. 10.1093/dnares/dsu018 24935866 PMC4195498

[mmi14420-bib-0026] Lai, D. , & Meyer, I. M. (2016). A comprehensive comparison of general RNA‐RNA interaction prediction methods. Nucleic Acids Research, 44, e61 10.1093/nar/gkv1477 26673718 PMC4838349

[mmi14420-bib-0027] Lalaouna, D. , Baude, J. , Wu, Z. , Toamisni, A. , Chicher, J. , Marzi, S. , … Moreau, K. (2019). RsaC sRNA modulates the oxidative stress response of *Staphylococcus aureus* during manganese starvation. Nucleic Acids Research, 47, 9871–9887. 10.1093/nar/gkz728 31504767 PMC6765141

[mmi14420-bib-0028] Lalaouna, D. , Carrier, M.‐C. , Semsey, S. , Brouard, J.‐S. , Wang, J. , Wade, J. T. , & Massé, E. (2015). A 3′ external transcribed spacer in a tRNA transcript acts as a sponge for small RNAs to prevent transcriptional noise. Molecular Cell, 58, 393–405. 10.1016/j.molcel.2015.03.013 25891076

[mmi14420-bib-0029] Lalaouna, D. , Desgranges, E. , Caldelari, I. , & Marzi, S. (2018). MS2‐affinity purification coupled with RNA sequencing approach in the human pathogen *Staphylococcus aureus* . Methods in Enzymology, 612, 393–411. 30502950 10.1016/bs.mie.2018.08.022

[mmi14420-bib-0030] Lalaouna, D. , Morissette, A. , Carrier, M.‐C. , & Massé, E. (2015). DsrA regulatory RNA represses both hns and rbsD mRNAs through distinct mechanisms in *Escherichia coli* . Molecular Microbiology, 98, 357–369. 26175201 10.1111/mmi.13129

[mmi14420-bib-0031] Lalaouna, D. , Prévost, K. , Eyraud, A. , & Massé, E. (2017). Identification of unknown RNA partners using MAPS. Methods, 117, 28–34. 10.1016/j.ymeth.2016.11.011 27876680

[mmi14420-bib-0032] Lu, P. , Wang, Y. , Zhang, Y. , Hu, Y. , Thompson, K. M. , & Chen, S. (2016). RpoS‐dependent sRNA RgsA regulates Fis and AcpP in *Pseudomonas aeruginosa* . Molecular Microbiology, 102, 244–259. 27381272 10.1111/mmi.13458

[mmi14420-bib-0033] Mandin, P. , & Gottesman, S. (2010). Integrating anaerobinc/aerobic sensing and the general stress response through the ArcZ small RNA. EMBO Journal, 29, 3094–3107. 20683441 10.1038/emboj.2010.179 PMC2944060

[mmi14420-bib-0034] Massé, E. , & Gottesman, S. (2002). A small RNA regulates the expression of genes involved in iron metabolism in *Escherichia coli* . Proceedings of the National Academy of Sciences, 99, 4620–4625. 10.1073/pnas.032066599 PMC123697 11917098

[mmi14420-bib-0035] Massé, E. , Vanderpool, C. K. , & Gottesman, S. (2005). Effect of RyhB small RNA on global iron use in *Escherichia coli* . Journal of Bacteriology, 187, 6962–6971. 10.1128/JB.187.20.6962-6971.2005 16199566 PMC1251601

[mmi14420-bib-0036] Melamed, S. , Peer, A. , Faigenbaum‐Romm, R. , Gatt, Y. E. , Reiss, N. , Bar, A. , … Margalit, H. (2016). Global mapping of small RNA‐target interactions in bacteria. Molecular Cell, 63, 884–897. 10.1016/j.molcel.2016.07.026 27588604 PMC5145812

[mmi14420-bib-0037] Miyakoshi, M. , Chao, Y. , & Vogel, J. (2015). Crosstalk between ABC transporter mRNAs via a target mRNA‐derived sponge of the GcvB small RNA. EMBO Journal, 34, 1478–1492. 25630703 10.15252/embj.201490546 PMC4474525

[mmi14420-bib-0038] Müller, P. , Gimpel, M. , Wildenhain, T. , & Brantl, S. (2019). A new role for CsrA: Promotion of complex formation between an sRNA and its mRNA target in *Bacillus subtilis* . RNA Biology, 16, 972–987. 31043113 10.1080/15476286.2019.1605811 PMC6546359

[mmi14420-bib-0039] Nielsen, J. S. , Lei, L. K. , Ebersbach, T. , Olsen, A. S. , Klitgaard, J. K. , Valentin‐Hansen, P. , & Kallipolitis, B. H. (2010). Defining a role for Hfq in Gram‐positive bacteria: Evidence for Hfq‐dependent antisense regulation in *Listeria monocytogenes* . Nucleic Acids Research, 38, 907–919. 10.1093/nar/gkp1081 19942685 PMC2817478

[mmi14420-bib-0040] Overlöper, A. , Kraus, A. , Gurski, R. , Wright, P. R. , Georg, J. , Hess, W. R. , & Narberhaus, F. (2014). Two separate modules of the conserved regulatory RNA AbcR1 address multiple target mRNAs in and outside of the translation initiation region. RNA Biology, 11, 624–640. 10.4161/rna.29145 24921646 PMC4152367

[mmi14420-bib-0041] Pain, A. , Ott, A. , Amine, H. , Rochat, T. , Bouloc, P. , & Gautheret, D. (2015). An assessment of bacterial small RNA target prediction programs. RNA Biology, 12, 509–513. 10.1080/15476286.2015.1020269 25760244 PMC4615726

[mmi14420-bib-0042] Papenfort, K. , Espinosa, E. , Casadesus, J. , & Vogel, J. (2015). Small RNA‐based feedforward loop with AND‐gate logic regulates extrachromosomal DNA transfer in Salmonella. Proceedings of the National Academy of Sciences of the United States of America, 112, 4772–4781. 10.1073/pnas.1507825112 PMC4553797 26307765

[mmi14420-bib-0043] Papenfort, K. , Sun, Y. , Miyakoshi, M. , Vanderpool, C. K. , & Vogel, J. (2013). Small RNA‐mediated activation of sugar phosphatase mRNA regulates glucose homeostasis. Cell, 153, 426–437. 10.1016/j.cell.2013.03.003 23582330 PMC4151517

[mmi14420-bib-0044] Prévost, K. , Salvail, H. , Desnoyers, G. , Jacques, J.‐F. , Phaneuf, E. , & Massé, E. (2007). The small RNA RyhB activates the translation of shiA mRNA encoding a permease of shikimate, a compound involved in siderophore synthesis. Molecular Microbiology, 64, 1260–1273. 10.1111/j.1365-2958.2007.05733.x 17542919

[mmi14420-bib-0045] Radoshevich, L. , & Cossart, P. (2018). Listeria monocytogenes: Towards a complete picture of its physiology and pathogenesis. Nature Reviews Microbiology, 16, 32–46. 10.1038/nrmicro.2017.126 29176582

[mmi14420-bib-0046] Robledo, M. , Schlüter, J.‐P. , Loehr, L. O. , Linne, U. , Albaum, S. P. , Jiménez‐Zurdo, J. I. , & Becker, A. (2018). An sRNA and cold shock protein homolog‐based feedforward loop post‐transcriptionally controls cell cycle master regulator CtrA. Frontiers in Microbiology, 9, 763 10.3389/fmicb.2018.00763 29740411 PMC5928217

[mmi14420-bib-0047] Rochat, T. , Bohn, C. , Morvan, C. , Le Lam, T. N. , Razvi, F. , Pain, A. … Bouloc, P. (2018). The conserved regulatory RNA RsaE down-regulates the arginine degradation pathway in Staphylococcus aureus. Nucleic Acids Res, 46, 8803–8816. 29986060 10.1093/nar/gky584 PMC6158497

[mmi14420-bib-0048] Romilly, C. , Caldelari, I. , Parmentier, D. , Lioliou, E. , Romby, P. , & Fechter, P. (2012). Current knowledge on regulatory RNAs and their machineries in *Staphylococcus aureus* . RNA Biology, 9, 402–413. 22546940 10.4161/rna.20103

[mmi14420-bib-0049] Romilly, C. , Lays, C. , Tomasini, A. , Caldelari, I. , Benito, Y. , Hammann, P. , … Vandenesch, F. (2014). A non‐coding RNA promotes bacterial persistence and decreases virulence by regulating a regulator in *Staphylococcus aureus* . PLoS Pathogens, 10, e1003979 10.1371/journal.ppat.1003979 24651379 PMC3961350

[mmi14420-bib-0050] Ruiz de los Mozos, I. , Vergara‐Irigaray, M. , Segura, V. , Villanueva, M. , Bitarte, N. , Saramago, M. , … Toledo‐Arana, A. (2013). Base pairing interaction between 5′‐ and 3′‐UTRs controls icaR mRNA translation in *Staphylococcus aureus* . PLoS Genetics, 9, e1004001 10.1371/journal.pgen.1004001 24367275 PMC3868564

[mmi14420-bib-0051] Saliba, A.‐E. , C Santos, S. , & Vogel, J. (2017). New RNA‐seq approaches for the study of bacterial pathogens. Current Opinion in Microbiology, 35, 78–87. 10.1016/j.mib.2017.01.001 28214646

[mmi14420-bib-0052] Smirnov, A. , Förstner, K. U. , Holmqvist, E. , Otto, A. , Günster, R. , Becher, D. , … Vogel, J. (2016). Grad‐seq guides the discovery of ProQ as a major small RNA‐binding protein. Proceedings of the National Academy of Sciences of the United States of America, 113, 11591–11596. 10.1073/pnas.1609981113 27671629 PMC5068311

[mmi14420-bib-0053] Tafer, H. , & Hofacker, I. L. (2008). RNAplex: A fast tool for RNA‐RNA interaction search. Bioinformatics, 24, 2657–2663. 10.1093/bioinformatics/btn193 18434344

[mmi14420-bib-0054] Toledo‐Arana, A. , Dussurget, O. , Nikitas, G. , Sesto, N. , Guet‐Revillet, H. , Balestrino, D. , … Cossart, P. (2009). The Listeria transcriptional landscape from saprophytism to virulence. Nature, 459, 950–956. 10.1038/nature08080 19448609

[mmi14420-bib-0055] Tomasini, A. , Moreau, K. , Chicher, J. , Geissmann, T. , Vandenesch, F. , Romby, P. , … Caldelari, I. (2017). The RNA targetome of *Staphylococcus aureus* non‐coding RNA RsaA: Impact on cell surface properties and defense mechanisms. Nucleic Acids Research, 45, 6746–6760. 10.1093/nar/gkx219 28379505 PMC5499838

[mmi14420-bib-0056] Uchiyama, I. (2006). Hierarchical clustering algorithm for comprehensive orthologous‐domain classification in multiple genomes. Nucleic Acids Research, 34, 647–658. 10.1093/nar/gkj448 16436801 PMC1351371

[mmi14420-bib-0057] Vecerek, B. , Moll, I. , Afonyushkin, T. , Kaberdin, V. , & Bläsi, U. (2003). Interaction of the RNA chaperone Hfq with mRNAs: Direct and indirect roles of Hfq in iron metabolism of *Escherichia coli* . Molecular Microbiology, 50, 897–909. 10.1046/j.1365-2958.2003.03727.x 14617150

[mmi14420-bib-0058] Voigt, K. , Sharma, C. M. , Mitschke, J. , Joke Lambrecht, S. , Voß, B. , Hess, W. R. , & Steglich, C. (2014). Comparative transcriptomics of two environmentally relevant cyanobacteria reveals unexpected transcriptome diversity. ISME Journal, 8, 2056–2068. 10.1038/ismej.2014.57 24739626 PMC4184020

[mmi14420-bib-0059] Waters, S. A. , McAteer, S. P. , Kudla, G. , Pang, I. , Deshpande, N. P. , Amos, T. G. … Tollervey, D. (2017). Small RNA interactome of pathogenic *E. coli* revealed through crosslinking of RNase E. EMBO Journal, 36, 374–387. 27836995 10.15252/embj.201694639 PMC5286369

[mmi14420-bib-0060] Wright, P. R. , Georg, J. , Mann, M. , Sorescu, D. A. , Richter, A. S. , Lott, S. , … Backofen, R. (2014). CopraRNA and IntaRNA: Predicting small RNA targets, networks and interaction domains. Nucleic Acids Research, 42, W119–W123. 10.1093/nar/gku359 24838564 PMC4086077

[mmi14420-bib-0061] Wright, P. R. , Mann, M. , & Backofen, R. (2018). Structure and interaction prediction in prokaryotic RNA biology. Microbiology Spectrum, 6 10.1128/microbiolspec.RWR-0001-2017 PMC11633574 29676245

[mmi14420-bib-0062] Wright, P. R. , Richter, A. S. , Papenfort, K. , Mann, M. , Vogel, J. , Hess, W. R. , … Georg, J. (2013). Comparative genomics boosts target prediction for bacterial small RNAs. Proceedings of the National Academy of Sciences of the United States of America, 110, E3487–3496. 10.1073/pnas.1303248110 23980183 PMC3773804

[mmi14420-bib-0063] Zheng, A. , Panja, S. , & Woodson, S. A. (2016). Arginine patch predicts the RNA annealing activity of Hfq from Gram‐negative and Gram‐positive bacteria. Journal of Molecular Biology, 428, 2259–2264. 10.1016/j.jmb.2016.03.027 27049793 PMC4884477

